# Exploring the limitations of language interpretation: A qualitative study on clinicians’ experiences at French Office of Immigration and Integration

**DOI:** 10.1371/journal.pgph.0002436

**Published:** 2023-12-18

**Authors:** Carter Brown, Guillaume Roucoux, Svetlane Dimi, Saleh Fahmi, Raj Banou Jeevan, Olivier Chassany, John Eric Chaplin, Martin Duracinsky

**Affiliations:** 1 Patient-Reported Outcomes Unit (PROQOL), UMR 1123, INSERM, Paris, France; 2 Pierre Louis Doctoral School of Public Health Sorbonne University, Paris, France; 3 Unité de Recherche Clinique en Economie de la Santé (URC-ECO) AP-HP Hôpital Hôtel-Dieu, Paris, France; 4 International Vaccination and Travel Medicine Center, Centre de Santé Familia Sol, Creil, France; 5 Institute of Health and Care Sciences The University of Gothenburg Centre for Person-Centred Care—GPCC Sahlgrenska Academy at Gothenburg University, Gothenburg, Sweden; 6 Service de Médecine Interne et d’Immunologie Clinique, AP-HP Hôpital Bicêtre, Le Kremlin-Bicêtre, France; University of Colorado Denver - Anschutz Medical Campus: University of Colorado - Anschutz Medical Campus, UNITED STATES

## Abstract

The concordance of communication between patients and health professionals is essential to promoting positive health outcomes. However, concordance may be broken where language barriers exist therefore creating a need to use interpretation services. This is the case when rapid diagnostic testing (RDT) of HIV, HBV, and HCV is offered to migrants. The use of interpreters to establish communication with patients having limited French proficiency (LFP) however, is often not used and can be problematic. Despite being offered, interpretation services are frequently underutilised, which makes communication challenging. This problem has not received enough attention in the literature, particularly in a technologically advanced setting where solutions may be found. Our objective was to explore how interpreters are used within the context of medical consultations when RDT for HIV, HBV, and HCV is offered to legal migrants with LFP. A cross-sectional qualitative study was used with a purposive sample that included doctors and nurses who had conducted rapid screening tests with migrants in four centers in France and who had access to interpretive services. Semi-structured interviews explored healthcare providers’ (HP) use of interpreters at the OFII. The use of professional or ad hoc interpreters, telephone interpreters, and the equivalence of concepts such as health literacy between the HP and the interpreter were explored. The utility of a new tool to promote communication concordance was evaluated. Twenty interviews were conducted with eleven doctors and nine nurses with a median age of 58 years (25–67 years). All participants had access to interpretive services although many did not solicit them because of 1) unawareness on how to use the services, 2) preconceived notions of the length of time to involve an interpreter and how this would add to consultation times, or 3) the proximity of an ad hoc interpreter. Not using interpreter services could result in RDTs not being offered to immigrants. Subjects such as confidentiality, the embarrassment of a third party’s presence, the lack of appropriate training and differing levels of health literacy were also discussed by participants. Insight from HPs allows us to better understand how both telephone and in-person interpretation are used, viewed, and why they are underused to communicate with limited French language skills patients. Our findings will help us develop a conceptual model for a digital communication tool to overcome barriers with migrant patients with limited French language skills.

## Introduction

Clear and coherent communication with patients including migrants is essential to providing high quality healthcare. Communication concordance between patients and providers has shown to reduce adverse health outcomes [1]. A major systematic review investigating the patient’s experience of communicating with primary care physicians found that the patient’s vulnerability increased due to language barriers with the healthcare provider (HP) [2]. In order to achieve communication concordance, formal and informal interpreters are often called upon to establish patient-provider communication. Although HPs or institutions may have access to formal, professional interpreters, they are often underutilised. Furthermore, research suggests that the availability of interpreter services does not decrease the use of *ad hoc*, in-person interpreters with foreign-speaking patients [3].

Informal interpreters, known as *ad hoc* interpreters, are individuals who are called upon to interpret despite a lack of training and qualification [4]. This could be a family or community member present during a consultation. *Ad hoc* interpreters have made critical mistakes due to the omission of information or misinterpretation which had inverse clinical impacts [5]. The same review found that the highest level of patient satisfaction was obtained when bilingual, over-the-phone professional interpreters were engaged while the lowest level of satisfaction was related to the use of *ad hoc* interpreters. Such interpreters can be in-person or over the phone.

Both clinicians and parents have at times relied on the children of migrant patients as *ad hoc* interprets. These *ad hoc* interpreters lack the proper training to deliver sensitive and confidential information, putting these individuals in difficult positions [6]. Neutral, qualitative, and confidential interpretative services are needed to create trust and productive exchanges in the patient-provider relationship. Stigmatization along with the lack of trust and confidentiality are difficulties reported by Latin Americans people living with HIV in a study investigating their experience with interpretive services [7]. Difficulties have also been reported by healthcare providers. Providers have noted a significant increase in consultation time needed when using interpretive services [8] along with the lack of general and sexual reproductive health knowledge, ultimately making it difficult to conduct consultations even in the presence of a professional interpreter [9].

We previously conducted a multicentric study at the French Office of Immigration and Integration (OFII) investigating the acceptability of optional rapid diagnostic tests (RDT) for HIV, hepatitis B virus (HBV), and hepatitis C virus (HCV) among migrants during compulsory medical consultations [10]. We demonstrated that migrants have a high level of acceptability of performing voluntary testing and yet these tests are often not performed. In nearly 30% of cases, communication barriers were reported as the main obstacle [11]. The mandatory consultations with migrants are an opportunity to provide screening to a generally vulnerable population however language discordance leads to missed opportunities. Therefore, access to improved interpretive services is perceived as a vital element in the delivery of optimal healthcare.

The objective of this study is therefore to explore how interpreters are used and perceived by HP during medical consultations when rapid screening for HIV, HBV, and HCV is offered to LFP legal migrants.

## Methods

### Study setting

This qualitative study was conducted between May 15th to October 20th, 2019 with contractual HPs conducting the mandatory medical check-ups in four OFII centers in France (Lyon, Nice, Cergy, and Montrouge). We targeted centres with different volumes of migrants, who employ more than five HPs and which provide services for people from different geographic locations.

### Population and sampling

An equal number of doctors and nurses, both males and females, were invited to participate using a purposive sampling method. Inclusion criteria was if the HP conducted rapid HIV, HBV, and HCV tests, had previously participated in the STRADA study, and had regular experience offering screening tests to migrants. We chose participants working for the same institution as they theoretically have access to the same resources.

### Data collection

In-depth interviews were conducted in French using an interview guide of opened-ended questions based on previous interviews with migrants on the same topic. They were recoded using a professional audio-recorder. Questions probed the nature of migrant medical visits, the HP’s point of view on the relevance of health literacy in communicating with migrants, the perceived experience of rapid testing of migrants, the creation of a new tool to promote rapid screening in the LFP population, and the use of telephone and informal interpreters.

In this paper we present data related to the HP’s point of view and experience with interpreters.

### Ethics

Study approval was obtained from Parisian Ethical Institutional Review Board (IRB n°00003835, protocol 2016/43NI) and registered with the French data protection agency, National Commission on Informatics and Liberty (CNIL n˚2008669). Approval was valid for the entire French territory. Strict measures of confidentiality have been taken to protect participants’ personal data. All methods were conducted in accordance to the Declaration of Helsinki.

## Results

### Data collection

Two researchers (SF, RBJ), one male and one female with backgrounds in public health, clinical medicine, and clinical psychology conducted key-informant interviews. The interviewers had no prior relationship with the participants thus, presented themselves and the objective of the study at the beginning of each interview. Interviews were conducted with eleven doctors and nine nurses who routinely conduct medical examinations at OFII. No participants refused to participate nor drop out of the study for any reason, at any time. Interviews lasted roughly 30 minutes (15–44 minutes) and were audio recorded. Informed consent from participants was obtained at the start of each audio recording of the interviews. Data were collected according to COREQ guidelines [12] respecting the participants confidentially. The two researchers shared their interview experiences and notes to determine when data saturation was achieved, which determined the final sample size. Interview recordings were transcribed verbatim by a transcription team (AH, FL, CB, RJ), thematically analysed using a general inductive approach [13]. Sonal software was used for coding by one researcher. The coding scheme was agreed upon by the field-work team, the data manager, and the scientific committee.

### Sociodemographic characteristics

Fourteen participants were females. Their median age was 58 years (25–67 years), with a median of 25 years of professional experience (2.5–40 years) and a median of three years at OFII (1 month– 22 years). Details are displayed in Table 1.

**Table 1:** HP sociodemographic characteristics.

	Participants
*N =*	20
*Sex*	
Male	6
Female	14
*Age (years)*	
20–29	1
30–39	1
40–49	3
50–59	8
60–69	5
*Localization*	
Center 1	6
Center 2	5
Center 3	8
Center 4	1
*Position*	
Medical doctor	10
Nurse	9
*With healthcare speciality* *	5
*Seniority as healthcare professional (in years)* *	
< 10	1
10–19	6
20–29	4
30–39	6
40–49	1
*Seniority at OFII (in years)* *	
< 1	3
1–9	9
10–19	4
≥ 20	2
*With another job outside of OFII job* *	15
*Previous professional experience (before OFII)* *	
With migrants	9
HIV/HBV/HCV prevention activities	7
*Native language* *	
French	15
Arabic	2
Russian	1
Malagasy	1
Fé’fé / Nufi (from Camaroon)	1
Cambodian	1
*Spoken language* *	
French	20
English	16
Spanish	4
Arabic	4
German	2
Russian	2
Portuguese	1
Malagasy	1
Fé’fé / Nufi (from Camaroon)	1
Cambodian	1

* Missing data for 2 participants.

### Thematic analysis

**Fig 1:**
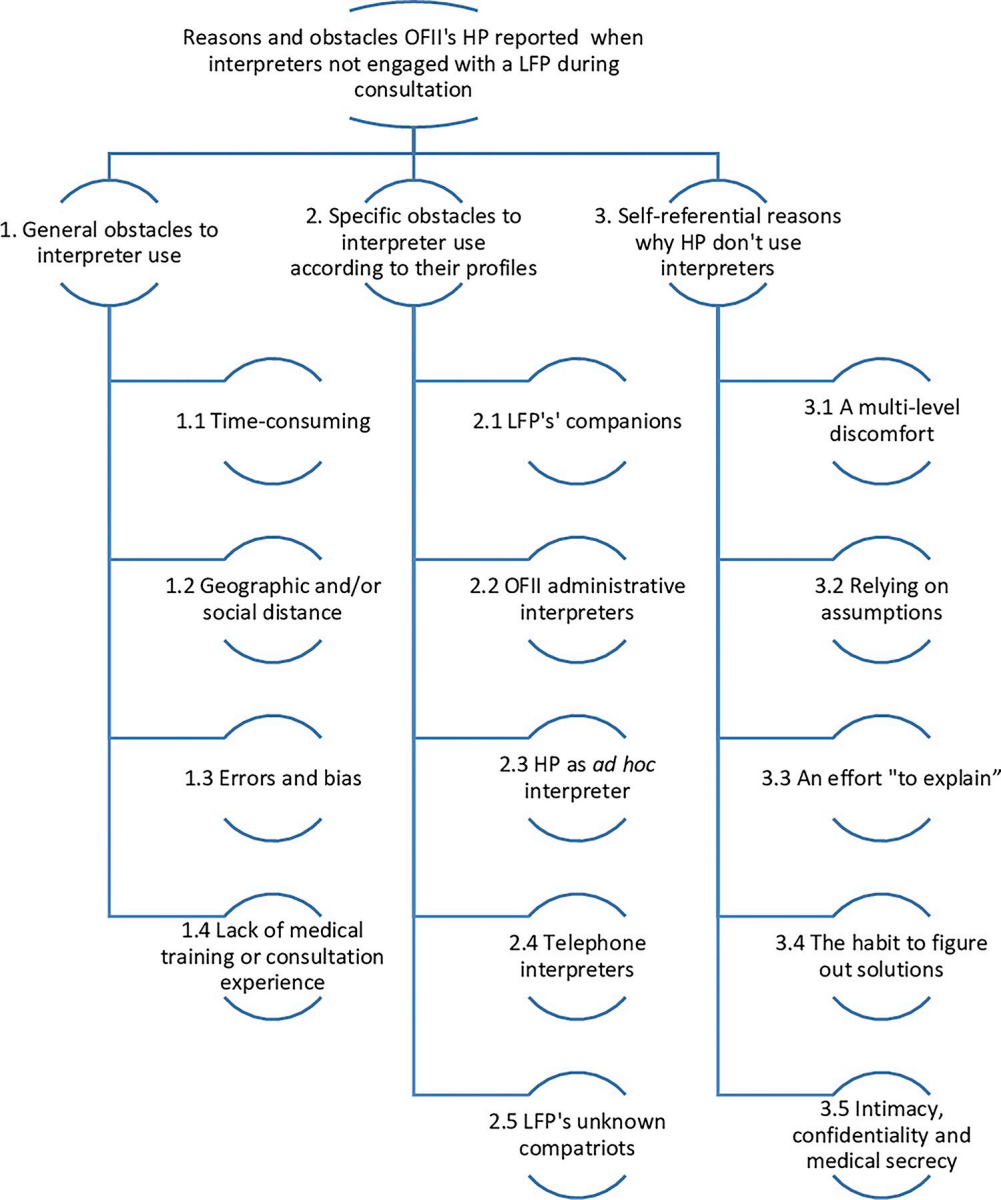
Thematic tree. Results of the thematic analysis of interviews.

Three major themes and 14 sub-themes were defined during the thematic analysis, detailed in the following diagram (Fig 1).

### General obstacles to the use of an interpreter

#### Time-consuming

All HPs but two nurses reported experience with interpreters (one MD did not respond). Regardless of the interpreter’s profile, interpretation was perceived as time-consuming, with the exception of one instance—with an OFII-trained interpreter available in-person. These instances were reported as time-consuming due to being put “on-hold” while waiting to be connected with the interpreter, significant amounts of information to be translated, specific syntax and vocabulary and/or the HPs perception of the interpreter’s willingness to work “well” and “efficient”.

#### Geographic and/or social distance

HPs mentioned seven different types of interpreters: migrants’ companions, OFII’s administration interpreters, migrant’s unknown compatriots present in the waiting room, other HPs/colleagues, telephone interpreters, professional interpreters hired by the migrant, and a "friend" of the migrant contacted by phone. According to their profiles, the physical presence and/or social distance of the interpreter, was the most salient factor in the choice of interpreter. In other words, HPs tended to select interpreters based on proximity (either to the patients in respect to their companions, or to their availability within the OFII) and not to professional status, linguistic skills or healthcare knowledge. According to the interpreter’s profiles most frequently solicited (Table 2), linguistic skills apart, interpreters’ geographical and/or social distance seemed to impact their use the most. In other words, HPs might favour geographical and social proximities (either the patient’s in respect to the companion, or theirs to the OFII’s administrative). Equally important, HPs preferences also depended largely on availability. Companions are present more often than other types of interpreters: "the entourage is very present here this morning (M, doctor)”. Geographical distance can be associated with a waste of time: "by the time I call [the telephone interpreting service], wouldn’t it be quicker to go and get the interpreter next door?" (F, nurse).

**Table 2:** Use of interpreter according to their profiles.

	Participants
*n =*	17
LFP’s companion	14
OFII administrative interpreter	8
LFP’s unknown compatriot	3
HP improvised-interpreter	1
Professional interpreter hired by the LFP	1
LFP’s “friend’ contacted by phone	1
Professional phone interpreter	1

#### Errors and bias: the filter question

Some HPs who were familiar with certain foreign languages found that interpreters, even professional ones, did not translate "*correctly*" and were often "*not neutral*" (F, nurse). One female doctor reported: "*I don’t speak Russian but I understand it*, *so [*…*] I realized that sometimes [the interpreter] didn’t translate correctly*, *and gave her personal opinion*.” A "*simple question*" became "*a whole speech*" or vice versa: *"[the interpreter] will speak [to the migrant] for five minutes when we asked a simple question*. *We wonder what he is saying*" (F, nurse). One doctor assumed that the “*filter*” (a term the researcher introduced during the interview) was systematic and inevitable, due to cultural or personal shared meanings between the interpreter and the patient. Another participant interpreted this notion as a source of confidence between the interpreter and the patient, which reassures the patient. It can be assumed that the companions were people who had been selected for their skills as an interpreter from the people that the patient had access to. Sometimes the interpreters were perceived as lacing rigor and precision by providing an answer that was not consistent with the question, or by supporting certain terms or ideas that the HP did not mention.

#### Lack of medical training or consultation experience

Another obstacle cited is that the interpreters are often not skilled enough to ask technical questions in the medical setting because "*the interpreter is not necessarily prepared*, *I think*, *to translate*" (F, doctor). Some technical terms, necessary for a precise clinical exam, may not be known by the interpreter: "*I asked the son to ask his mom*: *’does she have a cold*? *Does she have a sore throat and a cough*? *Does she have an ENT (ear*, *nose*, *and throat) infection*?*’ He asked his mom*, *’Are you sick*?*’ [laughter] I understood*, *I said*, *’No*, *that’s not what I asked*!" (M, doctor). Lack of medical training may explain interpretation mistakes

### Specific obstacles to interpreter use according to their profiles

#### LFP’s companions

When an interpreter was needed, HPs most often called upon the migrant’s companion for help. The accompanying person could be a family member (at large), a friend, or even an employer. HPs equally accepted the parents as the interpreter for the child, as they did the child as an interpreter for the parent. Assuming that in both cases the interpreter was aware of the patient’s health status. However, where a spouse was brought along as an interpreter they were not always accepted by the HP: for example where a husband accompanied a veiled woman he could be asked him to wait outside the consultation room. An employer was never accepted as an interpreter. A frequent problem encountered was the interpreter speaking on behalf of the patient, i.e. taking over the patient’s narrative and decision-making, thus reducing the patient’s autonomy in the consultation. One doctor described this situation as: "*the relative [*…*] responded in the person’s place*" (M, doctor). In such instances, HPs asked the companion to actually pose the question directly to the migrant. The presence of a companion also generated doubts in respect to the confidentiality and reliability of the information, in the eyes of the HPs.

### OFII administrative interpreters

Interpreters contacted directly by OFII were the “*the ideal case*” according to one female doctor. In many instances, these interpreters have already met the migrants during precedent administrative procedures, and may even "*offer [to translate during the medical consultation] because they knew that the person did not speak much*" (F, nurse). Such interpreters are external service providers and therefore require "additional resources” for the medical aspect of the migrant’s administrative process, which are not always available. Furthermore, interpreters have limits—several languages spoken by migrants were not spoken by the interpreters: "*We had [an interpreter] who spoke Chinese*, *but she is no longer here*" (F, nurse).

### HPs used as *ad hoc* interpreters

Often, HPs called on their fellow colleagues: “*Fortunately*, *we have a Russian speaker on staff*" (M, doctor). Some participants spoke of their experience improvising as the interpreter themselves: “*I went to the rescue for a nurse [*…*] I was the one who spoke English that day*" (F, doctor); another participant added, "*as I speak a little…gibberish*, *I was able to help out*" (F, nurse). This impacted the caregivers’ workload and productivity as they were called upon to translate, taking them away from their assigned tasks.

### Telephone interpreters

The majority of HPs knew they have access to telephone interpreters although only one participant had used it. A nurse, who had never used such service, perceived telephone interpreters useful if she should have to deliver news of a positive RDT result. The majority of participants expressed an eagerness to test this service. The reasons cited for non-use were diverse: no opportunity to do so, information about its existence only recently received, lack of know-how, perception of being complicated to use, or preference to try speaking English with the migrant. The service was mostly known for being time-consuming due to the lengthy procedure for getting in touch with an interpreter, and its inadequacy in terms of medical knowledge. It was considered highly expensive, may feel “*very impersonal*” (F, doctor) and put confidentiality at risk.

### LFP’s unknown compatriots

Three HPs spoke of compatriots, unknown to the LFP used as *ad hoc* interpreters: “*I look to see if there are other people of the same ethnicity during the medical visit*, *if there are people who can translate for me*, *if the person doesn’t mind*, *I bring in a third party*” (F, nurse). No obstacle was reported regarding this profile, they were seen as equally acceptable as professional interpreters hired by the migrant, or a "*friend*" contacted by phone (both cited once).

### Self-referential reasons HP had to not use interpreters

#### A multi-level discomfort

Various discomforts were expressed by participants. Most frequently, the HPs spoke of their personal discomfort during consultations with LFPs: "*I don’t like having someone [an interpreter] imposed upon me during consultations*" (M, doctor). One participant put themselves in the shoes of the migrant and the interpreter, speaking of the mutually perceived discomfort: "*He [the interpreter] feels that it’s impersonal too*. *[*…*] I wouldn’t necessarily like [to have an interpreter*, *if I were a migrant]*" (F, doctor). Another discomfort spoken of was confidentially in light of the interpreter’s presence: "*I think the person doesn’t want to share with another person the fact that they are going to be tested*" (F, doctor). **HP’s discomfort was associated with the patient’s intimacy, interpreters’ confidentiality and protection of medical secrecy**: "*I would not bring an interpreter to propose [sic] certain personal questions*" (F, nurse); "*We decided that we would not propose the test when there was the accompanying person [*…*] because it is a confidential test and afterwards to have to explain to the person we would have to call on the interpreter*" (F, nurse).

In respect to the interpreter’s presence to announce a positive RDT result, one participant added "*it’s not a question of respecting professional medical secrecy…we know that [the interpreter] is qualified*…*it’s more about delivering results of a diagnosis that [the migrant] didn’t expect*, *that they themselves didn’t take the initiative to show up to a screening center and ask for a telephone interpreter [to get screened]*" (F, doctor). Even if the HPs were unable to speak the patient’s language, many have asked the interpreter—regardless of their profile—to leave the room to spend a moment alone with the patient. Two strategies were used to provide confidential information without an interpreter: to speak French using with gestures or to use Google translate. Being alone with the patient allowed HP to talk about intimacy, the possibility of abuse, or to offer a RDT.

### Relying on assumptions

HPs spoke more often of their own perceptions over that of the patient’s, but they also frequently mentioned their colleagues’ experiences: "*from what colleagues told me*, *[using interpreters] takes time*" (M, doctor). Reiterating the notion of time, another participant said: "*I saw another doctor use [an interpreter] once*. *He stayed on the phone for I don’t know how long…*" (F, nurse).

Four mentioned their own preconceptions about patients, three of whom spoke of veiled women and their reluctance to talk about sexuality and further, to accept a RDT: "*We had asked ourselves about women with North African origins who were accompanied by their husbands*. *And we said to ourselves*: *’We’re going to get a hearty ’no’*, *whereas we have almost maximum acceptance*. *So*, *we sometimes have preconceived ideas [and therefore don’t propose the testing]*” (F, doctor). Gender seems to be important in relating to patients, and therefore, we can assume the gender of the interpreter would also play a role.

### An effort “to explain”

Participants spoke of the effort required to explain to a patient or a professional interpreter, what an RDT was or to explain a positive result once the patient had been screened. The latter situation seemed to be the more complicated dilemma for the HP to face: "*I think that [my explanation of the RDT] is even longer [than what I usually say when I offer a RDT to a French speaking migrant] because there is really*, *well I want to make everything clear in the sense that since there is a third party*, *the [migrant] must really understand that there is no connection with his or her administrative file*. *Even if I have to repeat it several times in different ways [laughs] so that it is really clear that even if this person is not from the medical field*, *he or she is still subject to confidentiality*. *This is why [we don’t offer the RDT in the absence of an interpreter] because what we were afraid of is a positive result and that we wouldn’t be able to explain to the patient*." (F, nurse).

### The habit to figure out solutions (“se débrouiller”)

Due to limited resources including the access to interpretation, HPs have learned to find resources that they can use as a last resort: "If I’m stuck, then I manage. So far, we’ve always managed, so we get by" (M, doctor); "I imagine it must be so complicated to get an interpreter [by telephone]. […] I prefer to get by with Google or with other people." (M, doctor).

## Discussion

Our research allows us to better understand how interpreters and interpretive services are both used and perceived by HP working at OFII who conduct medical visits with legal migrants.

Though each HP interviewed in this study has access to professional interpretive services, many were unaware of its existence or the modalities to use such services (Fig 1, 2.4). When interpreters were unavailable, some HP do not offer screening services therefore creating a missed testing opportunity (MTO) (Fig 1, 3.2). Furthermore, participants perceived the use of telephone interpreters with long telephone wait times and therefore, longer consultations. This coincides with quantitative data previously published demonstrating that over-the-phone interpreters were related to longer consultation times compared to in-person interpreters [14].

The confidential, comfortable environment where the patient could speak freely with their HP could be jeopardized by the presence of either in-person or over-the-phone interpreters. In our study, only one participant had used a professional, in-person interpreter in their practice although several participants associated the presence of an interpreter with a level of discomfort when discussing sensitive topics felt by the HP themselves, the patient, and even the interpreter even if they were professionally trained (Fig 1, 3.1, 3.5). Our research demonstrates that the majority of participants have relied on informal interpretive services provided by *ad hoc* interpreters such as people accompanying the LFP (relative, friend, spouse, etc.) (Table 1). An interpreter’s presence, especially when speaking about sensitive or culturally taboo topics can prohibit important conversations in the migrant-health provider relationship. Previous research which has investigated patients’ experiences with informal interpreters reported the patient’s feelings of guilt, embarrassment, and discomfort [15–17]. Thus, there are negative qualitative aspects of the use of interpreters in medical consultations with people with limited French language skills.

Refugee patients who reported language discordance with a HP demonstrated a high acceptability and usability of a digital communication assistance tool (DCAT) in Germany [18]. A digital, interpretive service could be a promising solution to address communication discordance in the patient-provider relationship in the Francophone context while responding to part of the need for more reliable, cost-effective, professional, and readily available interpretive service. Though we don’t image the complete elimination of interpreters from this context, we do believe that empowering the migrant by reducing the dependence on professional or *ad hoc* interpreters could decrease lengthy waiting-times, expensive services and equally importantly, discomforts associated with discussing sensitive topics related to HIV, HBV, and HCV risks. Overcoming such barriers could result in more qualitative consultations.

Healthcare providers expressed their desire to help their patients better understand RDT (3.3) and to overcome social and cultural distances (1.2) although preconceived notions of the patient in front of them at times inhibited their clinical judgement (3.2). A culturally trained interpreter would not simply interpret the doctor’s questions verbatim, but do so in a way that the patient will be able to understand. A keystone to patient-centered care is understanding the patient’s values and beliefs. It has been reported that physicians are poor judges of their patients’ health beliefs but when patient’s actively participant in consultations, physicians were able to better understand them [19]. A digital tool could be a reasonable solution to help empower patients by enabling them to play a more active role during consultations, helping healthcare providers better understand them although the translation should be adapted to the patient’s cultural.

### Recommendations

Further administrative and political efforts could be made to accommodate HPs healthcare who provide medical care to LFP migrants. For example, raising awareness whilst providing training to facilitate the HP’s access to existing interpretation services could incite HPs to call upon professional interpreters more frequently. Additional funding could be allocated to provide more in-person interpreters and to develop an innovative digital translation tool to promote screening. Missed testing opportunities in the migrant population create personal and public health problems for often already disadvantaged populations.

### External validity

Our findings could be true for other clinical settings where language barriers exists. Further research should be conducted to understand how both administrators and clinical experience and eventually, overcome language barriers in other clinical and non-clinical settings.

### Study strengths

We were able to directly interrogate HPs current practices and perceptions of interpretive solutions in light of our project to develop an application to overcome language barriers with translation and interpretive services. We were able to understand perceived barriers related to access and also the qualitative repercussions which interpreters could have on medical consultations with migrants. To our knowledge, this study is the first of its kind in the Francophone context.

### Limitations

This study was limited to HPs working at OFII and therefore is not representative of all HPs’ experiences with interpretive services in France. Furthermore, only one participant had used formal, interpretive services. No male nurse participated in this study, but there aren’t any identifiable reasons to believe that gender would demonstrate different results. The overall qualitative STRADA study included migrants, but no interpreters. To complete the picture of human interpretive services, a further study should include interpreters to report their own experiences during medical consultations with people with limited French language skills, considering their unique position between patients and HP.

## Conclusion

Although professional interpretive services are available to each HP, we found that they are widely underutilized and system-wide, awareness and working knowledge is lacking. HP most frequently rely upon *ad hoc* interpreters which, although convenient, introduce complexities in the patient-provider relationship. A campaign to raise awareness of existing interpretive services could help prevent MTO whilst a digital communication tool could be more accessible and user-friendly solution in the near future.

Our research provides us with a better understanding of current practices and obstacles that HPs experience related to interpreters, including their perceptions–whether formal or informal. This knowledge will be used to inform the development of an innovative, digital application in the ApiDé study [20] which aims to validate a communication application in an attempt to address language barriers and ultimately, increase rapid diagnostic testing rates of HIV, HBV, and HCV in the limited French proficiency population in France.

## Supporting information

S1 File Interview guide–French. (DOCX)

S2 File Interview guide–English. (DOCX)
