# Supplementary material for: Exploring the limitations of language interpretation: A qualitative study on clinicians’ experiences at French Office of Immigration and Integration
Source: PLOS Glob Public Health. 2023 Dec 18;3(12):e0002436. doi: 10.1371/journal.pgph.0002436 (PMC10727366; doi:10.1371/journal.pgph.0002436)
Supplement: S1 File — (DOCX) [file pgph.0002436.s001.docx]

*Interview guide – French*

Nous souhaitons réaliser des entretiens avec les soignants au sein de l’OFII pour connaître votre perception et vos expériences sur les barrières linguistiques qui se présentent pendant la visite médicale et l’offre des TROD chez les migrants non francophones. On cherche aussi à générer une liste de phrases clefs qui seront utilisés pour la motivation des migrants pour accepter de faire les TROD. Ensuite ces phrases seront traduites en plusieurs longues pour être utilisé avec les migrants non francophones.

Avec votre permission, j’enregistrerai. Celui-ci sera ensuite retranscrit par écrit pour tout en gardant l’anonymat complet.

1. Acceptez-vous de participer à cet entretien et acceptez-vous qu’il soit enregistré ?
2. Avant de commencer, est-ce que vous avez des questions sur cet entretien ?

Tout d’abord j’ai quelques questions générales :

1. ***Données sociodémographiques (à compléter directe sur la fiche)***
2. Puis-je savoir votre âge ?
3. Quel est votre spécialité médicale ?
4. Depuis combien de temps exercez-vous votre profession ?
5. Depuis combien de temps travaillez-vous dans cette DT ? et avec l’OFII en général ?
6. Depuis combien de temps travaillez-vous avec des populations migrantes ?
7. Quelle activité exercez-vous en dehors de l’OFII ?
   1. Est-ce que vous avez déjà travaillé dans un service réalisant spécifiquement la prévention ou la prise en charge du VIH ou des hépatites ?
8. Quelle est votre langue maternelle ? Parlez-vous d’autres langues ? à quel niveau ? Pouvez-vous par exemple lire de matériel scientifique et des brochures dans une autre langue (Anglais)
9. ***Avez-vous déjà participé à l’étude Qualitative faites avant le début de STRADA ?***

***Oui / Non***

1. ***Cette partie est pour comprendre vos interactions avec les non francophones en dehors de l’OFII***
2. Avez-vous dans votre entourage personnel des personnes non francophones ?
3. Comment communiquez-vous avec eux ?

***Si il/elle exerce une autre activité professionnelle en dehors de l’OFII***

1. Dans votre activité professionnelle en dehors de l’OFII, est ce que vous rencontrez des migrants ou des personnes non francophones ?
2. Comment vous faites pour communiquer avec eux ?
3. Voyez-vous de différences entre cette communication et celle pendant votre travail à L’OFII ?
4. Lesquelles ?
5. Quels outils de traduction connaissez-vous ?

**Relance** (applications / site / autres)

1. Avez-vous jamais utilisé un outil de traduction en dehors de l’OFII ?
2. Lesquels ?
3. Avez-vous jamais demandé à quelqu’un de vous faire la traduction ?
4. Qui ?

**Relance :** interprète Professional ? un ami ? Membre de famille ?

1. ***Maintenant on va passer à la partie concernant les visites médicales avec des migrants non francophones à l’OFII***
2. Comment vous faites pour connaitre le niveau de compréhension de français du migrant

**Relance,** sur quels critères se baser vous ?

1. Pouvez-vous le décrire les outils que vous utilisez pour communiquer avec le migrant non francophone?

**Relance**: Les classeurs ? Les brochures ? Google traduction ? Autres ?

1. Dans quel cas décidez-vous d’appeler l’accompagnant pour faire la traduction ?
2. Vous pensez que la présence de l’accompagnant peut modifier les réponses du patient ?

***Si oui,***

1. Merci de nous donner un exemple ?
2. Pouvez-vous nous dire en terme de minutes et /ou pourcentage la différence entre la durée de visite avec migrant non francophone et francophone ?
3. Et donc si la consultation avec patient non francophone est souvent plus longue comment faites-vous pour trouver le temps pour couvrir toutes les thématiques importantes ?
4. ***Maintenant on va passer à la partie concernant les tests des TROD avec des migrants non francophones***
5. Dans votre équipe, quelle est la personne qui propose les TROD au migrants ?
6. Donc, est ce que vous en avez proposé?
7. Est-ce que ça vous est déjà arrivé une fois où vous avez pensé de proposer les TROD mais vous ne l’avez pas fait ?

***Si oui,***

1. Pourquoi?
2. Selon vous la personne aurait pu refuser ?
3. Est-ce que ça vous est déjà arrivé que le migrant accepte de faire les TROD et puis change d’avis après la visite médicale ?
4. Pensez-vous c’est dû à quoi ?

***Si oui,***

1. Est-ce que ça vous est déjà arrivé que le migrant refuse de faire les TROD et puis change d’avis après la visite médicale ?

***Si oui,***

1. Pensez-vous c’est du à quoi ?
2. Décrivez-moi comment vous faites pour proposer les TROD à un migrant non francophone ?
3. Pensez-vous que la barrière linguistique est différente pour l’offre de dépistage TROD que pour la visite médicale ?

***Si oui,***

1. Merci de nous donner un exemple
2. Trouvez-vous que vous pouvez utiliser le même outil de traduction pour l’offre du dépistage TROD, que pour les VM ?

***Si non,***

1. Quelles différences existent alors?
2. Comment les spécificités culturelles des migrants peuvent modifier la façon dont vous offrez de dépistage ?
3. Les résultats de l’étude qualitative déjà réalisée au début du projet STRADA ont montré que la difficulté d’aborder les sujets sensibles comme la sexualité et l’usage de drogue existent plutôt chez le soignant que chez les migrants, qu’est-ce que vous en dites ?
4. Est-ce qu’il vous arrive d’éviter ce sujet avec un migrant parce qu’il ne parle pas Français ? Pensez-vous que le fait d’aborder des sujets sensibles en présence d’un traducteur peut gêner les patients ?
5. Est-ce que cela vous gêne vous comme médecin /infirmière ?

***Si oui***

1. Dans ce cas quelle sera la solution pour éviter d’avoir un traducteur selon vous ?
2. Connaissez-vous combien de langues y a-t-il pour les questionnaires TROD screen ?
3. Que pensez-vous de la traduction des TROD screen ?
4. Aviez-vous vu un migrant non francophone qui a eu un problème avec ses traductions ?
5. Quelles questions ?
6. Avez-vous annoncé ou assister à l’annonce d’un résultat positif de TROD à un migrant non francophone ?
7. Comment s’est passé ?
8. Quelles difficultés avez-vous rencontrées ?
9. ***Cette partie concerne l’usage des interprètes par téléphones (ISM)***
10. Connaissez-vous le service d’interprète par téléphone proposé pour l’OFII ?
11. Avez-vous utilisé ce service (ISM)?

***Si non,***

1. Ça vous intéresse de l’essayer ? Pourquoi ?
2. On voudrait avoir faire une évaluation de ce service plus exacte avec votre retour plus détaillé seriez-vous d’accord de faire un test, par exemple d’utiliser l’ISM systématiquement pendant une semaine donnée ?

***Si oui,***

1. Qu’est ce vous en pensez ? Est-ce que vous avez eu des problèmes pour l’utiliser ?
2. Utiliserez-vous ce service encore une fois ?
3. Selon vous qu’est-ce qu’on peut faire pour rendre ce service plus efficace ?
4. Est-ce qu’utiliser l’ISM pour l’offre des TROD est différente que celle avec la visite médicale ?
5. Dans quels aspects ?
6. ***On passe pour la dernière partie sur le concept de la Health Literacy***

***« La littératie en santé » représente les connaissances, la motivation et les compétences permettant d’accéder, comprendre, évaluer et appliquer de l’information dans le domaine de la santé ; pour ensuite se forger un jugement et prendre une décision en terme de soins de santé, de prévention et de promotion de la santé, dans le but de maintenir et promouvoir sa qualité de vie tout au long de son existence » [Sørensen ; 2012].***

1. Pensez-vous que ce niveau de littératie en santé peut affecter la compréhension et l’acceptation de l’migrant concernant l’offre de dépistage TROD ?
2. Si oui décrivez-moi comment pourriez-vous mesurer ce niveau ?
3. Pensez-vous que connaitre le niveau « La littératie en santé » du migrant peut influencer votre façon de présenter le dépistage ?
4. Si oui, comment ?
5. ***En fin on voudrai prendre votre opinion sur la création d’un nouvel outil pour aider à la promotion du dépistage aux patient non francophones***
6. D’après ce que vous avez vu, quelle est le côté le plus accrocheur des TROD qui fait que les migrants sont intéressés par le faire ?
7. Pour les migrants non francophone ça sera quoi les phrases clefs, les plus indispensables, à traduire pour les rendre intéressé par les TROD ?
8. Pensez-vous que selon le profil du migrant la motivation pour un dépistage change?

**Si oui,**

1. Les phrases utilisées doivent elles changer aussi ? par exemple ?
2. Imaginer que je suis un patient non francophone, ça sera quoi les phrases que vous aimeriez avoir traduites pour mieux me faire comprendre l’importance du dépistage ?
3. Comment imaginer vous un outil qui peut vous aider à mieux expliquer l’importance du dépistage TROD ? **Relance** (Dessins ? Photo ? Pictogrammes ? vidéo ? autres)
