# Supplementary material for: Exploring the limitations of language interpretation: A qualitative study on clinicians’ experiences at French Office of Immigration and Integration
Source: PLOS Glob Public Health. 2023 Dec 18;3(12):e0002436. doi: 10.1371/journal.pgph.0002436 (PMC10727366; doi:10.1371/journal.pgph.0002436)
Supplement: S2 File — (DOCX) [file pgph.0002436.s002.docx]

*Interview guide – English*

We would like to conduct interviews with health care providers at OFII to learn about your perceptions and experiences with language barriers that arise during the medical visit and the offering of TRODs to non-French speaking migrants. We also aim to generate a list of key phrases that will be used to motivate migrants to accept the TROD. These phrases will then be translated into several languages for use with non-French speaking migrants.

With your permission, I will record. This will then be transcribed into writing while maintaining complete anonymity.

1. Do you agree to participate in this interview and do you agree to have it recorded?

2. Before we begin, do you have any questions about this interview?

First of all I have some general questions:

1. ***Socio-demographic data (to be completed directly on the form)***
2. May I know your age?
3. What is your medical specialty?
4. How long have you been in practice?
5. How long have you been working in this DT? and with OFII in general?
6. How long have you been working with migrant populations?
7. What professional activity do you carry out outside of OFII?
   1. Have you ever worked in a service that specifically provides HIV or hepatitis prevention or care?
8. What is your first language? Do you speak any other languages? At what level? For example, can you read scientific materials and brochures in another language (English)?
9. ***Have you already participated in the Qualitative study done before the start of STRADA?***

***Yes / No***

1. ***This part is to understand your interaction(s) with non-French speakers outside OFII***
2. Do you have any non-Francophones in your personal circle?
3. How do you communicate with them?

***If he/she has another professional activity outside OFII***

1. In your professional activity outside OFII, do you meet migrants or non-French speaking people?
2. How do you communicate with them?
3. Do you see any difference between this communication and the one during your work at OFII?
4. Which ones?
5. What translation tools do you know?

**Repeat, if need** (applications / site / others)

1. Have you ever used a translation tool outside of OFII?
2. Which ones?
3. Have you ever asked someone to translate for you?
4. Who did you ask?

**Exmple:** Professional interpreter? Family member?

1. ***Maintenant on va passer à la partie concernant les visites médicales avec des migrants non francophones à l’OFII***
2. How do you know the migrant's level of understanding of French?

Based on what criteria?

1. Can you describe the tools you use to communicate with the non-French speaking migrant?

**Example**: Binders? Brochures? Google translation? Other?

1. When do you decide to call the companion to do the translation?
2. Do you think that the presence of the companion can modify the patient's answers?

***If yes, please specify***

1. Please give us an example?
2. Can you tell us in terms of minutes and/or percentage the difference between the duration of the visit with a non-French speaking migrant and a French speaking one?
3. If the consultation with a non-French speaking patient is often longer, how do you manage to find the time to cover all the important topics?
4. ***Now we will move on to the part concerning the testing of TROD with non-French speaking migrants***
5. In your team, which person offers the TROD to migrants?
6. Have you offered any?
7. Have you ever thought about offering the TRODs but didn't?

***If yes, please specify***

1. Why is this?
2. In your opinion, could the person have refused?
3. Has it ever occurred that the migrant agrees to do the TROD and then changes his or her mind after the medical visit?
4. What do you think caused this?

***If yes, please specify***

1. Have you ever had a migrant refuse to take the TROD and then change his mind after the medical examination?

***If yes…***

1. What do you think it is due to?
2. Describe how you go about offering TROD to a non-French speaking migrant.
3. Do you think the language barrier is different for offering TROD testing than for the medical visit?

***If yes…***

1. Please give us an example
2. Do you find that you can use the same translation tool for the TROD testing offer, as for VM?

***If no…***

1. What differences exist then?
2. How might the cultural specificities of migrants change the way you offer testing?
3. The results of the qualitative study already carried out at the beginning of the STRADA project showed that the difficulty of approaching sensitive subjects such as sexuality and drug use exists more among the caregiver than among the migrants, what do you say about this?
4. Do you ever avoid this subject with a migrant because he or she does not speak French? Do you think that talking about sensitive subjects in the presence of a translator can make patients uncomfortable?
5. Does it bother you as a doctor/nurse?

***If yes…***

1. If so, what would be the solution to avoid having a translator in your opinion?
2. Do you know how many languages there are for the TROD screen questionnaires?
3. What do you think about the translation of the TROD screen?
4. Have you seen a non-French speaking migrant who had a problem with the translations?
5. What questions?
6. Have you announced or witnessed the announcement of a positive TROD result to a non-French speaking migrant?
7. How did it go?
8. What difficulties did you encounter?
9. ***This section concerns the use of telephone interpreters (ISM)***
10. Are you familiar with the telephone interpreter service offered for OFII?
11. Have you used this service (ISM)?

***If no…***

1. Are you interested in trying it? Why or why not?
2. We would like to have a more accurate evaluation of this service with your more detailed feedback. Would you be willing to do a test, for example to use the ISM systematically during a given week?

***If yes…***

1. What do you think? Did you have any problems using it?
2. Will you use this service again?
3. What do you think can be done to make this service more effective?
4. Is using the ISM to offer TRODs different than using the medical visit?
5. In what aspects?
6. ***Moving on to the final section on the concept of Health Literacy
   "Health literacy "represents the knowledge, motivation and skills to access, understand, evaluate and apply information in the field of health; to then form a judgment and make a decision in terms of health care, prevention and health promotion, with the aim of maintaining and promoting his quality of life throughout his life" [Sørensen; 2012].***
7. Do you think this level of health literacy may affect the migrant's understanding and acceptance of the TROD testing offer?
8. If so, describe how you would assess this level?
9. Do you think knowing the migrant's level of health literacy can influence how you present the screening?
10. If so, how?
11. ***Finally, we would like to ask your opinion on the creation of a new tool to help promote screening to non-French speaking patients***
12. From what you have seen, what is the most eye-catching aspect of TROD that makes migrants interested in doing it?
13. What would be the most important key phrases for non-French speaking migrants to translate to make them interested in the TROD?
14. Do you think that depending on the profile of the migrant, the motivation for testing changes?

**If yes…**

1. Do the sentences used have to change too? Can you give an example?
2. Imagine that I am a non-French speaking patient, what phrases would you like to have translated to make me understand the importance of screening better?
3. How would you imagine a tool that could help you better explain the importance of TROD testing? (Drawings? Photo? Pictograms? Video? Other)
